# Suspected parental gonadal/gonadosomatic mosaicism for a TINF2 mutation in two sisters with dyskeratosis congenita

**DOI:** 10.3389/fgene.2026.1833814

**Published:** 2026-07-15

**Authors:** Tao Xie, Hanying Nong, Jiali Jiang, Mengxin Yang, Jialiang Liao, Zhihao Lin, Yuping Li, Bobo Xie, Hongying Wei

**Affiliations:** 1 Department of Pediatrics, The Second Affiliated Hospital of Guangxi Medical University, Nanning, China; 2 State Key Laboratory of Targeted Oncology, Guangxi Medical University, Nanning, China

**Keywords:** bone marrow failure, dyskeratosis congenita, functional exploration, suspected gonadal/gonadosomatic mosaicism, telomere dysfunction, TINF2 gene

## Abstract

**Background:**

Dyskeratosis congenita (DC; OMIM: 127550) is a rare inherited bone marrow failure syndrome. *TINF2* mutations are the second most common genetic cause of DC, and most cases arise from *de novo* mutations. Although the *TINF2* p.Thr284Pro variant has been reported in isolated cases, its pathogenic role has not been functionally validated, and its potential association with suspected parental gonadal/gonadosomatic mosaicism has not been previously described.

**Objective:**

To evaluate the functional impact of the *TINF2* p.Thr284Pro variant and explore its association with suspected parental gonadal/gonadosomatic mosaicism in a DC pedigree in which two affected sisters were born to clinically unaffected parents. The findings may provide evidence for improved molecular diagnosis and genetic counseling in DC.

**Methods:**

Clinical and genetic investigations were performed in a family suspected of DC. Multi-tissue sequencing was conducted in the parents and the proband. To evaluate the functional consequences of the variant, wild-type and p.Thr284Pro mutant *TINF2* overexpression plasmids were constructed and transfected into HEK293T cells. TINF2 protein expression was analyzed by Western blotting. Cell proliferation was assessed using the CCK-8 assay, telomere length was measured by quantitative PCR, and cellular senescence was evaluated using SA-β-galactosidase staining.

**Results:**

Both affected sisters exhibited an incomplete classical DC phenotype, characterized primarily by pancytopenia and nail dystrophy. Genetic analysis identified the same heterozygous *TINF2* c.850A>C (p.Thr284Pro) variant in both patients. This variant was absent in peripheral blood and other parental tissues (oral mucosa and hair follicles), suggesting an apparently *de novo* occurrence in the siblings and raising the possibility of suspected parental gonadal/gonadosomatic mosaicism. Functional assays provided preliminary supportive evidence that the mutant construct was associated with lower detected TINF2 protein levels compared with wild type (*P* = 0.01), decreased cellular proliferation suggestive of impaired proliferative capacity, a reduced relative telomere length signal (*P* < 0.001), and increased senescence-associated β-galactosidase activity. These findings provide preliminary evidence regarding the potential functional impact of the *TINF2* p.Thr284Pro variant.

**Conclusion:**

This study provides the first clinical-genetic evidence for suspected parental gonadal/gonadosomatic mosaicism of the TINF2 p.Thr284Pro variant, along with exploratory *in vitro* data supporting its potential functional impact. The affected sisters exhibited an incomplete classical DC phenotype (nail dystrophy without reticular skin pigmentation or oral leukoplakia), thereby expanding the clinical spectrum of DC and highlighting important implications for genetic counseling.

## 1 Introduction

Dyskeratosis congenita (DC) is a rare inherited disorder, with an estimated incidence of approximately 1 in 1,000,000 individuals. The classical clinical triad consists of reticular skin pigmentation, oral leukoplakia, and nail dystrophy (Jose et al., 2018; Zhang et al., 2025). As the disease progresses, patients often develop life-threatening complications such as bone marrow failure (BMF), along with an increased risk of malignancies and multi-organ involvement, particularly pulmonary and hepatic complications—including liver fibrosis, cirrhosis, and portal hypertension—with neurological involvement occurring in specific phenotypes (Zhang et al., 2025; Dokal et al., 2015; Vittal et al., 2023; Uria-Oficialdegui et al., 2023).

DC most commonly presents in childhood and exhibits diverse genetic inheritance patterns, namely, X-linked recessive, autosomal dominant, and autosomal recessive (Savage, 2022). The underlying pathological mechanism of DC involves defective telomere maintenance, leading to critically shortened telomeres. To date, 20 pathogenic genes have been identified, including *DKC1, TINF2, TERC, TERT*, and *RTEL1*. Approximately 80% of reported mutations directly or indirectly affect telomerase activation or the assembly of telomere-protective complexes (Savage, 2022; Goldman et al., 2005; Walne and Dokal, 2009). When telomeres shorten to a critical length, cells approach the Hayflick limit—the maximum number of cell divisions a cell can undergo before entering irreversible growth arrest—resulting in impaired proliferation, cellular senescence, apoptosis, and genomic instability. Together, these processes contribute to progressive tissue and organ failure (Lansdorp, 2022; Mó et al., 2022). Among known DC-associated genes, *TINF2* mutations are the second most common cause of DC, accounting for approximately 11% of all cases, second only to DKC1 (20%–25%), and most cases arise from *de novo* mutations (Dokal et al., 2015; Savage, 2022; Alter et al., 2012). The TIN2 protein, encoded by *TINF2*, is a core component of the shelterin complex—a structure that safeguards telomeres. The double-stranded telomeric DNA-binding proteins TRF1 and TRF2 require TIN2 to form a stable complex with TPP1/POT1, which regulates telomere length homeostasis (Dokal et al., 2015; Ye et al., 2004). *TIN2* has a dual regulatory role: it stabilizes TRF1, a negative regulator of telomere elongation (Ye and de Lange, 2004), while also facilitating telomerase recruitment through TPP1, enabling telomere extension and maintenance (Takai et al., 2017; Hu et al., 2017). Compared with heterozygous mutations in TERT or TERC, which encode telomerase components, *TINF2* variants are typically *de novo*, and are associated with earlier onset, shorter telomeres, and more severe clinical phenotypes (Alter et al., 2012; Savage et al., 2008; Walne et al., 2008; Norris et al., 2021).

Despite advances in understanding the molecular basis of DC, the disease remains highly heterogeneous both clinically and genetically, complicating genotype–phenotype correlation and diagnostic precision (Savage, 2022; Tejero et al., 2025). Although the *TINF2* p.Thr284Pro variant has previously been reported in a small number of cases (Gupta et al., 2017), functional assays have not been available. Here, we investigate a unique DC family in which two sisters presented with manifestations of pancytopenia (pallor, petechiae, and ecchymoses) and nail dystrophy but lacked the typical skin pigmentation or oral leukoplakia seen in classical DC. Born to phenotypically normal parents, both siblings carry the identical *TINF2* c.850A>C (p.Thr284Pro) variant. This study aims to evaluate the functional impact of this variant and explore its association with suspected parental gonadal/gonadosomatic mosaicism through genetic analysis and *in vitro* functional assays, thereby providing evidence for improved molecular diagnosis and genetic counseling in DC.

## 2 Materials and methods

### 2.1 Ethics approval

This study was conducted in strict accordance with the principles of the Declaration of Helsinki and was approved by the Ethics Committee of the Second Affiliated Hospital of Guangxi Medical University (Approval No. 2025-KYL (079)). Written informed consent, including permission for the publication of relevant images and clinical details, was obtained from the legal guardians of all participating children.

### 2.2 Clinical data collection and laboratory evaluation

Clinical data were retrospectively collected from January 2017 to September 2025 at the Department of Pediatrics, Second Affiliated Hospital of Guangxi Medical University. The study focused on a suspected DC family that included two affected female siblings (the proband and her elder sister) who met the criteria for familial verification. The following clinical and laboratory data were collected and analyzed: sex, age at disease onset, family history, major clinical manifestations (nail dystrophy, reticular skin pigmentation, and oral leukoplakia), laboratory findings (complete blood count, bone marrow morphology, flow cytometric analysis of bone marrow CD34^+^ cells, and TRF-based telomere length assessment), genetic testing results (peripheral blood, oral mucosal cells, and hair follicles from the proband and parents; peripheral blood only was available from the elder sister), treatment history, and follow-up outcomes.

### 2.3 Genetic testing and analysis

Peripheral blood samples were collected from all four family members (4 mL from the proband; 2 mL each from the parents and elder sister) using EDTA anticoagulant tubes. In addition, oral mucosal cell samples and hair follicle samples were obtained from the proband (Patient 2) and both parents. All samples were analyzed at Kangxu Medical Laboratory (Beijing, China) using a custom telomere-related gene panel (including *TINF2*) for next-generation sequencing (NGS). The average sequencing depth of the *TINF2*-targeted region was 125× in the proband (Patient 2) and 119× in Patient 1 (elder sister). Variants identified as potentially pathogenic were verified by Sanger sequencing. Variant annotation and clinical significance were assessed using OMIM, HGMD, and ClinVar databases; common polymorphisms were filtered out using gnomAD and 1,000 Genomes databases. Pathogenicity predictions were performed using MutationTaster, Provean, and PolyPhen-2 software. Variant classification followed the ACMG guidelines.

### 2.4 Terminal restriction fragment (TRF) analysis

Peripheral blood samples (2 mL each, EDTA-anticoagulated) were collected from the proband and both parents, and TRF analysis was performed by KingMed Diagnostics (Shanghai, China).

Average telomere length was measured using the terminal restriction fragment (TRF) assay, the classical gold-standard method for quantitative telomere length assessment (Kimura et al., 2010; Yu et al., 2024). Genomic DNA was digested with HinfI/RsaI restriction endonucleases, separated by agarose gel electrophoresis, transferred by Southern blotting, and hybridized with telomere-specific probes to accurately determine the average absolute telomere length (kb) of the cell population.

### 2.5 Flow cytometric analysis of bone marrow CD34^+^ hematopoietic stem cells

Bone marrow aspirate samples from the proband (3 mL, EDTA-anticoagulated) were collected and analyzed by Wuhan Kangshengda Medical Laboratory using flow cytometry. Fluorescently labeled anti-CD34 monoclonal antibodies and isotype controls were used for cell surface staining according to standard protocols. Data were acquired using a flow cytometer. The gating strategy was as follows: a CD45/SSC gate was first applied to select CD45^+^ and CD45^+^dim cells (P2), excluding debris; CD34^+^ low-SSC cells were then gated as P3 on a CD34/SSC plot displaying P2; cells were further refined by selecting CD45dim cells (P4) and those with higher FSC (P5). CD34^+^ cells were ultimately defined as the intersection of gates P2, P3, P4, and P5.

### 2.6 *In vitro* functional validation

A plasmid-based overexpression model was used to assess the functional impact of the *TINF2* p.Thr284Pro variant on telomere function, cell proliferation, and senescence in 293T cells.

#### 2.6.1 Plasmid construction, cell culture, and transfection

To evaluate the functional impact of the *TINF2* p.Thr284Pro variant, three recombinant plasmids were constructed by GentleGen Biotechnology (Suzhou, China):OE-WT (wild-type *TINF2* overexpression vector), OE-Thr284Pro (mutant *TINF2* p.Thr284Pro overexpression vector), and OE-CTRL (empty control vector). All plasmids were cloned into the pCDNA3.1 (+) backbone using NheI/EcoRI restriction sites and contained an ampicillin resistance gene. Correct insertion was confirmed by Sanger sequencing and restriction digestion. Plasmid quality met standard criteria (endotoxin <0.1 EU/µg, A_260_/A_280_ = 1.95, concentration = 1.06 μg/μL) and the plasmids were stored at −20 °C until use. 293T cells (IM-H222, Yimo Biotechnology, Xiamen, China) were cultured in DMEM high-glucose medium (Gibco, Cat. No. C11965500BT) supplemented with 10% fetal bovine serum (Gibco, Cat. No. 10099141) and 1% penicillin-streptomycin (Biosharp, Cat. No. BL505 A) at 37 °C in a 5% CO_2_ incubator. Exponentially growing cells (80% confluency) were seeded into 6-well plates (5 × 10^5^ cells/well) and transfected with the above plasmids using Lipofectamine™ 2000 according to the manufacturer’s protocol.

#### 2.6.2 Western blot analysis of TINF2 protein expression

Following transfection, cells were washed three times with pre-chilled PBS and lysed on ice for 15 min using RIPA lysis buffer. Lysates were centrifuged at 12,000×g for 30 min at 4 °C, and protein concentrations were determined using the BCA method. Equal amounts of protein were subjected to SDS-PAGE (4%–20% HEPES-Tris gels; 150 V, 60–80 min) and transferred to PVDF membranes via wet transfer. Membranes were blocked in 5% non-fat milk in TBS for 1 h at room temperature or overnight at 4 °C. Primary antibodies used were mouse anti-FLAG (Proteintech, Cat. No. 66008-4-Ig; 1:30,000), mouse anti-GAPDH (Proteintech, Cat. No. 60004-1-Ig; 1:20,000), and rabbit anti-TIN2 (HUABIO, Cat. No. HA722432; 1:1,000). After washing with TBST, membranes were incubated with HRP-conjugated goat anti-mouse secondary antibody (1:20,000) for 40 min at room temperature. Signals were visualized using ECL substrate, imaged with a Tanon 5,200 system, and quantified by Image-Pro Plus 6.0, with GAPDH serving as the internal control.

#### 2.6.3 CCK-8 assay for cell proliferation

Cell proliferation was evaluated using a CCK-8 assay. 293T cells were seeded into 96-well plates (5 × 10^3^ cells/well) and incubated for 0, 24, 48, and 72 h under standard conditions. At each time point, the culture medium was replaced with 100 µL of CCK-8 working solution (MedChemExpress, Cat. No. HY-K0301) and incubated for 4 h at 37 °C in the dark. Absorbance was measured at 450 nm using a microplate reader.

#### 2.6.4 Quantitative PCR analysis of relative telomere length

Genomic DNA was extracted from cells in each experimental group using an RNA/DNA Extraction Kit (Beyotime, Cat. No. R0017 S) according to the manufacturer’s instructions. DNA concentration and purity were assessed by ultraviolet spectrophotometry (A_260_/A_280_ = 1.8–2.0). DNA samples were diluted to 5 ng/μL. Quantitative PCR was performed using SYBR Green chemistry on a QX300 Real-Time PCR System (Sichuan Jielaimei Technology Co., Ltd.). The reaction mixture (20 μL) was shown in Table 1. PCR conditions were as follows: initial denaturation at 95 °C for 10 min, followed by 40 cycles of denaturation at 95 °C for 15 s and annealing/extension at 60 °C for 1 min. Three technical replicates were performed for each sample. 36B4 (RPLP0) served as the internal reference. Relative telomere length (T/S ratio) was calculated using the 2^−ΔΔCt^ method.

**TABLE 1:** Real-time PCR reaction mix for telomere length measurement.

Reagents	Volumes (μl)	Final concentration
SYBR green master mix (2×)	10	1×
Primer F (2 μM)	1	0.1 μM
Primer R (2 μM)	1	0.1 μM
ddH2O	4	​
DNA (5 ng/μL)	4	20 ng total

Primer sequences were as follows:

Telomere forward:

5′-CGGTTTGTTTGGGTTTGGGTTTGGGTTTGGGTTTGG GTT-3’

Telomere reverse:

5′-GGCTTGCCTTACCCTTACCCTTACCCTTACCCTTAC CCT-3’

36B4 forward: 5′-ACTGGTCTAGGACCCGAGAAG-3’.

36B4 reverse: 5′-TCAATGGTGCCTCTGGAGATT-3’.

#### 2.6.5 SA-β-gal staining for cellular senescence

Forty-eight hours post-transfection, senescence-associated β-galactosidase activity was assessed using a β-galactosidase staining kit (Yeasen, Cat. No. 40754ES60) following the manufacturer’s instructions. Five random fields per group were captured under a light microscope (scale bar = 100 μm), and SA-β-Gal-positive cells were quantified as the percentage of total cells (positive/total × 100%).

### 2.7 Statistical analysis

All statistical analyses were performed using SPSS 25.0 (IBM, Armonk, NY, USA). Graphs were generated with GraphPad Prism 8.0, and protein band intensities were quantified with Image-Pro Plus 6.0. Data with normal distribution are expressed as mean ± standard deviation (mean ± SD; x̄ ± s). Comparisons between two groups were performed using independent-samples t-tests. Comparisons among multiple groups were performed using one-way analysis of variance (ANOVA) followed by Tukey’s honestly significant difference (HSD) *post hoc* test. Non-normally distributed data are expressed as median (P25, P75) and compared using the Mann-Whitney U (two groups) or Kruskal–Wallis H (multiple groups) tests. All experiments were performed with three independent biological replicates. A two-sided *P* < 0.05 was considered statistically significant.

## 3 Results

### 3.1 Clinical and genetic findings in the affected family

**FIGURE 1:**
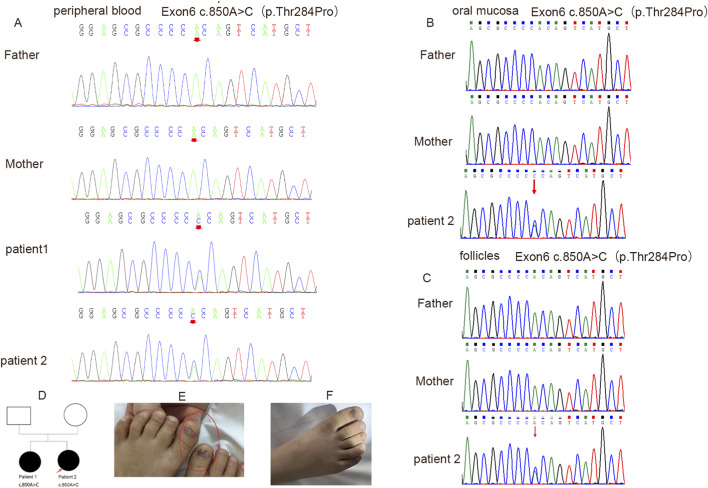
Genetic analysis and clinical characteristics of an apparently *de novo TINF2* c.850A>C (p.Thr284Pro) variant in a family with dyskeratosis congenita Sanger sequencing of the proband’s family revealed a heterozygous missense variant *TINF2* c.850A>C (p.Thr284Pro) in peripheral blood (red arrows). **(A)** Both affected siblings (patient 1 and patient 2) carried the variant, while their parents showed only the wild-type allele. **(B,C)** Oral mucosa and hair follicle samples from the father and mother were wild-type, whereas those from the proband showed the same heterozygous variant. **(D)** Pedigree of the dyskeratosis congenita (DC) family: squares denote males, circles denote females; filled symbols represent affected individuals carrying the c.850A>C (p.Thr284Pro) variant. The red arrow indicates the proband (patient 2). **(E,F)** Both affected siblings presented with typical toenail dystrophy. Abbreviations: DC, dyskeratosis congenita; F, father; M, mother.

In a Chinese family affected by DC, two female children were identified as heterozygous carriers of a *TINF2* c.850 A>C (p.Thr284Pro) variant. Both parents were clinically healthy, non-consanguineous, and had no family history of DC (NM_001099274.3:c.850 A>C, Figure 1D).

#### 3.1.1 Clinical characteristics

Both patients presented with pancytopenia and aplastic anemia (AA). Physical examination revealed pallor, petechiae, ecchymoses, and nail dystrophy (Figures 1E,F), but neither child exhibited the reticular skin pigmentation or oral leukoplakia characteristic of classical DC.

Patient 1 (elder sister, proband’s sibling): She developed pancytopenia at age 2 years. At age three, she was admitted with fever and abdominal pain and was diagnosed with DC. Complete blood count (CBC) showed leukocytes at 2.2 × 10^9^/L, absolute neutrophil count 0.54 × 10^9^/L, hemoglobin 48.1 g/L, and platelets 1 × 10^9^/L. Bone marrow morphology revealed markedly decreased trilineage hematopoiesis, consistent with aplastic anemia.

**FIGURE 2:**
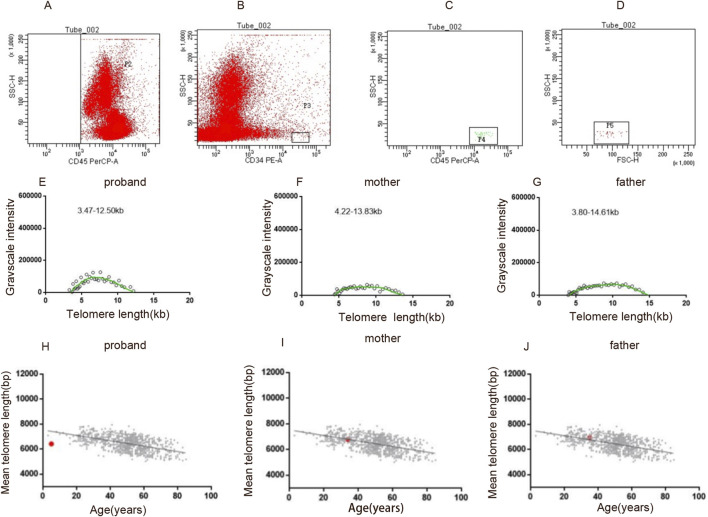
Severe reduction of hematopoietic stem cells and shortened telomeres in the proband. **(A–D)** Flow cytometry demonstrated a marked reduction in CD34^+^ hematopoietic stem cells in the proband’s bone marrow. **(E–G)** Telomere length distribution in peripheral blood was analyzed by terminal restriction fragment (TRF) assay for the proband **(E)**, mother **(F)**, and father **(G)**. **(H–J)** Mean telomere length in the proband was below the normal range for age, whereas both parents’ values were within age-appropriate limits. Reference telomere lengths: 7.28–7.58 kb for a 5-year-old child; 6.67–6.96 kb for a 34-year-old adult; 6.64–6.94 kb for a 35-year-old adult. Telomere length was measured using the TRF (terminal restriction fragment) analysis method.

Patient 2 (proband): She developed pancytopenia at age 3 years. At age five, she presented with cough, fever, and pancytopenia, and was diagnosed with DC. CBC revealed leukocytes 1.6 × 10^9^/L, absolute neutrophil count 0.42 × 10^9^/L, hemoglobin 43 g/L, and platelets 1 × 10^9^/L. Bone marrow morphology showed severe hypocellularity with markedly reduced granulocytic, erythroid, and megakaryocytic precursors, consistent with AA. Flow cytometry demonstrated a significant decrease in CD34^+^ hematopoietic stem cells (0.04% of leukocytes), indicating impaired hematopoiesis (Figures 2A–D).

#### 3.1.2 Genetic analysis

##### 3.1.2.1 Variant identification and validation

Sanger sequencing confirmed that both affected sisters carried a heterozygous TINF2 c.850 A>C (p.Thr284Pro) variant in peripheral blood, while both parents showed only the wild-type allele (Figure 1A). Further analysis of oral mucosa and hair follicle tissues confirmed the absence of the variant in both parents, whereas the proband carried the mutation in the corresponding tissues (Figures 1B,C). The presence of the same variant in two affected siblings, together with negative somatic testing in both parents, provides key evidence consistent with suspected parental gonadal/gonadosomatic mosaicism (Figure 1). To assess the pathogenic potential of this variant, population databases (gnomAD, 1,000 Genomes, dbSNP) were screened, confirming that this variant is extremely rare and not a common polymorphism. Although the TINF2 p.Thr284Pro (c.850 A>C) variant has been reported previously, its potential functional impact has not been evaluated *in vitro* (Gupta et al., 2017). In silico predictions were inconsistent: MutationTaster indicated “pathogenic,” PolyPhen-2 predicted “possibly damaging,” while Provean classified it as “neutral.” According to the 2015 ACMG/AMP guidelines, this variant was preliminarily classified as “Likely Pathogenic” (Supplementary Table S1).

#### 3.1.3 Telomere length and additional genetic testing

Telomere restriction fragment (TRF) analysis of the proband’s peripheral blood revealed a telomere length range of 3.47–12.50 kb (mean 6.42 kb), shorter than the age-matched reference range. The father’s telomere length ranged from 3.80–14.61 kb (mean 6.94 kb), and the mother’s from 4.22–13.83 kb (mean 6.79 kb), both within normal limits (Figures 2E–J). The proband’s karyotype was 46,XX without abnormalities. Comet assay demonstrated DNA damage in lymphocytes, although chromosomal aberration rates were normal.

### 3.2 Clinical outcome

Patient 1 died at 3.5 years of age while awaiting hematopoietic stem cell transplantation. Patient 2 is currently alive and remains on the transplant waiting list, receiving regular supportive care (Table 2). To clarify its functional impact, *in vitro* experiments were performed.

**TABLE 2:** Clinical characteristics of family members.

Parameter	Patient 1	Patient 2	Mother	Father
Gender	Female	Female	Female	Male
Age at first symptoms	2 y	3 y	None	None
Age at diagnosis	3 y	5 y	None	None
*TINF2* variant	c.850A>C (p.Thr284Pro)	c.850A>C (p.Thr284Pro)	Wild type	Wild type
Telomere length status	-	Shortened (6.42 kb)	Normal (6.79 kb)	Normal (6.94 kb)
Mucocutaneous features				
Nail dystrophy	Present	Present	Absent	Absent
Reticular skin pigmentation	Absent	Absent	Absent	Absent
Oral leukoplakia	Absent	Absent	Absent	Absent
Phenotype classification	Incomplete classical DC	Incomplete classical DC	Healthy	Healthy
Pancytopenia	Present	Present	Absent	Absent
Hematologic parameters				
WBC	2.2 × 10^9^/L	1.6 × 10^9^/L	5.66 × 10^9^/L	11.93 × 10^9^/L
ANC	0.54 × 10^9^/L	0.42 × 10^9^/L	2.74 × 10^9^/L	6.24 × 10^9^/L
Hemoglobin (g/L)	48.1	43	122	132
PLT	1 × 10^9^/L	1 × 10^9^/L	310 × 10^9^/L	219 × 10^9^/L
Bone marrow findings	Markedly reduced trilineage hematopoiesis; consistent with AA	Severely hypocellular marrow; trilineage hypoplasia; consistent with AA	-	-
Remaining systemic symptoms/signs				
Growth/Development	Normal	Normal	Normal	Normal
Dental abnormalities	None	None	None	None
Ophthalmologic	Normal, no retinal abnormalities or epiphora	Normal, no retinal abnormalities or epiphora	-	-
Neurological	Normal	Normal	-	-
Hepatic	Normal	Normal	-	-
Pulmonary	Normal	Normal	-	-
Gastrointestinal	Normal	Normal	-	-
Treatment	Supportive care; awaiting HSCT	Supportive care; awaiting HSCT	-	-
Outcome	Deceased at 3 y^6^ months (BMF complications)	Alive; awaiting HSCT	Healthy	Healthy

Abbreviations: AA, aplastic anemia; BMF, bone marrow failure; DC, dyskeratosis congenita; HSCT, hematopoietic stem cell transplantation; y, years; WBC, white blood cell count; ANC, absolute neutrophil count; Hb, hemoglobin; PLT, platelet count. Note: —, not applicable/not tested. Reference ranges: WBC, 5–12 × 10^9^/L; ANC, 2.50–8.40 × 10^9^/L; Hb, 120–140 g/L; PLT, 125–350 × 10^9^/L. Reference telomere lengths: 7.28–7.58 kb for a 5-year-old child; 6.67–6.96 kb for a 34-year-old adult; 6.64–6.94 kb for a 35-year-old adult. Telomere length was measured using the TRF (terminal restriction fragment) analysis method.

### 3.3 Functional characterization of the *TINF2* p.Thr284Pro variant

#### 3.3.1 Protein expression (western blot)

**FIGURE 3:**
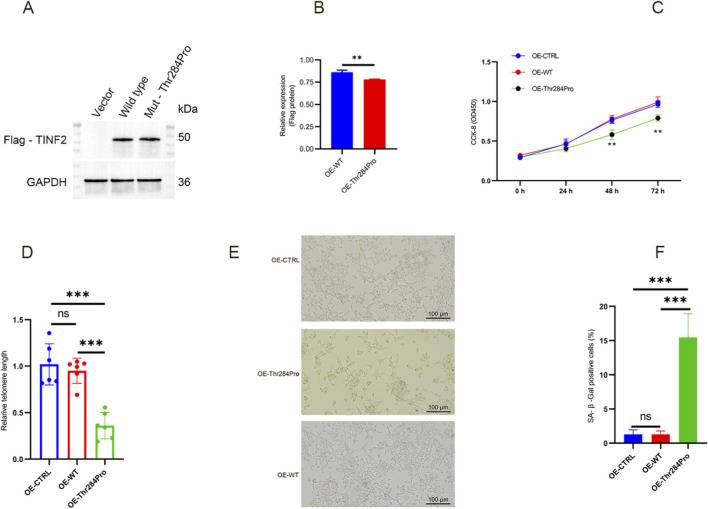
Functional Consequences of the *TINF2* p.Thr284Pro Variant: Telomere Dysfunction and Senescence-Associated β-Galactosidase Activity. **(A)** Western blot analysis showed lower detected TINF2 protein levels in the OE-Thr284Pro group compared with OE-WT in this overexpression system. Representative blots are shown (*TINF2* detected using anti-FLAG antibody; GAPDH as control). **(B)** Quantitative analysis showed significantly lower detected TINF2 protein levels in OE-Thr284Pro cells compared with OE-WT (*P* = 0.01). **(C)** Cellular proliferative activity was markedly reduced in OE-Thr284Pro cells at 48 and 72 h compared with OE-WT and OE-CTRL (*P* < 0.01). **(D)** qPCR analysis showed a reduced relative telomere length signal (T/S ratio) in the OE-Thr284Pro group compared with the OE-WT and OE-CTRL groups (*P* < 0.001). **(E)** Representative SA-β-Gal staining images showed an increased number of blue-stained cells in the OE-Thr284Pro group (scale bar: 100 μm). **(F)** Quantitative analysis of SA-β-Gal staining. The percentage of SA-β-Gal-positive cells was significantly higher in the OE-Thr284Pro group than in the OE-WT and OE-CTRL groups (*P* < 0.001). Note: No significant differences were observed between OE-WT and OE-CTRL (P > 0.05). Data are presented as mean ± standard deviation from three independent biological replicates. Statistical analyses were performed using one-way ANOVA followed by Tukey’s HSD *post hoc* test. Ns, not significant; *P < 0.05; **P < 0.01; ***P < 0.001. Abbreviations: OE-CTRL, empty vector control; OE-WT, wild-type *TINF2* overexpression vector; OE-Thr284Pro, mutant *TINF2* (p.Thr284Pro) overexpression vector. P_1_, OE-CTRL vs. OE-WT; P_2_, OE-CTRL vs. OE-Thr284Pro; P_3_, OE-WT vs. OE-Thr284Pro.

To evaluate the impact of the *TINF2* p.Thr284Pro variant on protein expression, FLAG-tagged wild-type and mutant constructs were overexpressed in 293T cells, with GAPDH used as an internal control. Western blot analysis showed that the mutant construct was associated with lower detected TINF2 protein levels compared with the wild-type construct in this overexpression system (*P* = 0.01) (Figures 3A,B). Original uncropped blot images are provided in Supplementary Figure S1. To exclude potential confounding by differential transfection efficiency, TINF2 protein was further assessed using an anti-TINF2 antibody. TINF2 levels were significantly lower in the OE-Thr284Pro group (1.55 ± 0.03) than in the OE-WT group (1.71 ± 0.04) (*P* = 0.013) (Supplementary Figure S2), consistent with the FLAG result.

#### 3.3.2 Cell proliferative activity (CCK-8 assay)

Cellular proliferative activity was monitored over 72 h post-transfection. CCK-8 assay results showed that OD_450_ values increased continuously over time (0–72 h) in all groups, indicating time-dependent increases in cellular proliferation. The OE-Thr284Pro group exhibited significantly lower OD_450_ values at 48 h and 72 h than the OE-WT and OE-CTRL groups (*P* < 0.01), suggesting impaired cellular proliferation. No significant difference was detected between OE-WT and OE-CTRL groups (P > 0.05) (Table 3; Figure 3C).

**TABLE 3:** Cellular proliferation of 293T cells at 0–72 h after transfection (CCK-8 assay).

Parameters	OE-CTRL	OE-WT	OE-Thr284Pro	P_1_	P_2_	P_3_
OD450 (0 h)	0.29 ± 0.02	0.32 ± 0.02	0.30 ± 0.02	0.09	0.68	0.17
OD450 (24 h)	0.47 ± 0.06	0.46 ± 0.04	0.40 ± 0.04	0.92	0.17	0.19
OD450 (48 h)	0.76 ± 0.04	0.78 ± 0.05	0.58 ± 0.06	0.72	0.005	0.003
OD450 (72 h)	0.97 ± 0.04	0.99 ± 0.07	0.79 ± 0.04	0.55	0.005	0.002

OE-CTRL, empty vector control; OE-WT, wild-type *TINF2* overexpression vector; OE-Thr284Pro, mutant *TINF2* (p.Thr284Pro) overexpression vector. Values represent absorbance at 450 nm (OD_450_). Data are presented as mean ± standard deviation from three independent biological replicates. Statistical analysis was performed using one-way ANOVA followed by Tukey's HSD post *hoc* test. P_1_, OE-CTRL vs. OE-WT; P_2_, OE-CTRL vs. OE-Thr284Pro; P_3_, OE-WT vs. OE-Thr284Pro. *P* < 0.05 was considered statistically significant.

#### 3.3.3 Telomere length (qPCR)

Relative telomere length assessed by qPCR showed a reduced relative telomere length signal in cells expressing the *TINF2* p.Thr284Pro mutant compared with those expressing wild-type or OE-CTRL constructs (*P* < 0.001), while no difference was found between the OE-WT and OE-CTRL groups (*P* = 0.53) (Figure 3D).

#### 3.3.4 SA-β-gal staining

SA-β-Gal staining revealed a markedly increased number of blue-stained cells in the OE-Thr284Pro group compared with the OE-WT and OE-CTRL groups under microscopy (Figure 3E), which was confirmed by quantitative analysis (*P* < 0.001). No significant difference was observed between the OE-WT and OE-CTRL groups (*P* = 0.99) (Figure 3F).

## 4 Discussion

In the present study, two female siblings with DC were found to carry the same apparently *de novo* heterozygous TINF2 c.850 A>C (p.Thr284Pro). Multi-tissue sequencing of both parents revealed no evidence of somatic mosaicism within the detection limit of Sanger sequencing and provided key genetic evidence consistent with suspected parental gonadal/gonadosomatic mosaicism. Further *in vitro* analyses provided preliminary supportive evidence that the mutant construct was associated with lower detected TINF2 protein levels in this overexpression system and that this variant may induce telomere dysfunction, which may contribute to increased senescence-associated β-galactosidase activity and impaired cellular proliferation. To our knowledge, this is the first report linking the *TINF2* p.Thr284Pro variant to suspected parental gonadal/gonadosomatic mosaicism in a DC family. These findings contribute to a better understanding of the potential functional impact underlying this variant.

The *TINF2* c.850 A>C (p.Thr284Pro) variant identified in this family exhibited an apparently *de novo* recurrence pattern among siblings, consistent with previous observations that most DC-associated *TINF2* variants arise as spontaneous *de novo* events (Walne et al., 2008). Genetic analysis showed that both parents were wild-type across multiple tissues, while both siblings carried the same variant, consistent with an apparently *de novo* presentation and suspected parental gonadal/gonadosomatic mosaicism (Xu et al., 2016). This inheritance pattern is compatible with the concept of mosaicism, defined as the presence of genetically distinct cell populations within an individual derived from a single zygote (Thorpe et al., 2020). The mutation may be confined to a subset of the parents’ germ cells, making it undetectable in somatic tissue sequencing and complicating the assessment of recurrence risk. The pattern observed here, with both parents testing wild-type and both children affected, is most consistent with suspected parental gonadal/gonadosomatic mosaicism (Xu et al., 2016; Thorpe et al., 2020). Early, accurate detection of such mosaicism is critical for optimizing genetic testing and counseling. However, this remains technically challenging, as conventional Sanger sequencing cannot reliably detect low-level mosaicism (<10–15%), and mosaicism may be limited to germ cells (Manderstedt et al., 2020). Because suspected parental gonadal/gonadosomatic mosaicism increases the complexity of recurrence risk evaluation for *de novo* mutations (Myers et al., 2018; Jónsson et al., 2018), traditional tests may fail to detect low-level mosaicism. Advanced technologies such as ultra-deep sequencing and droplet digital PCR (ddPCR) could clarify mosaic ratios, estimate the timing of mutation events, and improve counseling accuracy (Saura et al., 2023; Domogala et al., 2021; Jamuar and Walsh, 2014). Although direct gonadal sampling (e.g., testicular biopsy or ovarian aspiration) poses ethical and technical challenges (Breuss et al., 2020), and mosaic states may differ between peripheral blood and germ cells (Frisk et al., 2022; Chen et al., 2023), these high-sensitivity methods offer a viable approach to enhancing detection and diagnostic accuracy. Clinically, both patients developed symptoms in early childhood (ages 2 and 3 years, respectively) with severe bone marrow failure (BMF) as the main feature. The age at onset was younger than the median age (5 years) reported for Chinese patients with DC (Li et al., 2019), consistent with the early onset and rapid progression characteristic of *TINF2*-related DC (Alter et al., 2012; Savage et al., 2008; Walne et al., 2008). Interestingly, both patients presented only with nail dystrophy, without reticular skin pigmentation or oral leukoplakia, representing an incomplete classical DC phenotype. This finding aligns with previous reports showing that only 37% of patients with DC exhibit the full diagnostic triad, whereas approximately 10% exhibit none of the classic diagnostic features (Ward et al., 2018) The heterogeneity of DC’s clinical and genetic features, together with its rarity, complicates genotype–phenotype correlation (Savage, 2022; Tejero et al., 2025). Studies in Chinese cohorts reported a median onset at 5 years but a median diagnosis age of 16 years, indicating significant diagnostic delay (Li et al., 2019). Patients with characteristic mucocutaneous symptoms are more readily recognized, which may explain why many DC cases in China are initially identified by dermatologists (Li et al., 2019). Notably, the same *TINF2* c.850 A>C (p.Thr284Pro) variant was previously reported by Gupta et al. in twins with Revesz syndrome—a severe DC subtype characterized by exudative retinopathy, intracranial calcifications, cerebellar hypoplasia, and severe bone marrow failure (Gupta et al., 2017). In striking contrast, patients in the present family exhibited an incomplete classical DC phenotype without retinal abnormalities or intracranial involvement. This marked difference highlights the broad clinical spectrum of DC, whereby the same variant may lead to phenotypes ranging from severe syndromic manifestations to milder incomplete classical forms. Clinically, *TINF2*-related DC should remain a diagnostic consideration even in patients lacking the complete diagnostic triad or severe syndromic manifestations. In genetic counseling, the same parental gonadal/gonadosomatic mosaic variant may result in phenotypes spanning any point along the DC clinical spectrum in future pregnancies and may not necessarily resemble the phenotype of the proband.

Terminal restriction fragment (TRF) analysis in this study confirmed shortened telomeres in the proband’s blood (3.47–12.50 kb, mean 6.42 kb), while both parents had age-appropriate telomere lengths (father: 3.80–14.61 kb, mean 6.94 kb; mother: 4.22–13.83 kb, mean 6.79 kb). These results are consistent with telomere dysfunction induced by the TINF2 c.850 A>C (p.Thr284Pro) variant, which leads to telomere shortening, a hallmark of DC (Khincha et al., 2017). This also provides key laboratory evidence explaining the proband’s early and severe BMF. Further analysis showed a normal karyotype (46,XX), excluding chromosomal abnormalities as the cause of BMF. Comet assay of the proband’s lymphocytes showed DNA damage but a normal chromosomal aberration rate (Li et al., 2015), suggesting that the mutation induces DNA damage via telomere dysfunction rather than by disrupting chromosome structure. These findings reinforce that telomere attrition is the central mechanism driving DC pathology (Zhang et al., 2025; Karremann et al., 2020).

Previous research has shown clear population heterogeneity in DC-related genes (Callea et al., 2022). International data indicate that mutations in DKC1, TERC, TERT, NOP10, NHP2, and *TINF2* account for about 60% of DC cases, involving X-linked recessive, autosomal dominant, and autosomal recessive patterns (Dokal et al., 2015; Savage and Bertuch, 2010; Dokal et al., 2022). In China, reported cases mainly involve mutations in *DKC1*, *TERT*, and *TINF2* (Li et al., 2019), suggesting population-specific differences in the genetic spectrum. The identification of the *TINF2* c.850 A>C (p.Thr284Pro) variant in this study further expands the mutational spectrum of DC in the Chinese population and provides a reference for TINF2 screening. Given the clinical heterogeneity, diagnostic delay, and genetic diversity of DC, strict adherence to diagnostic criteria, standardized testing (including telomere length assessment and multi-tissue sequencing), and strengthened differential diagnosis are essential to reduce missed diagnoses and misdiagnosis. Furthermore, the clinical course of this family highlights several key principles regarding hematopoietic stem cell transplantation (HSCT) management in patients with DC. First, early molecular diagnosis is crucial for timely initiation of transplant evaluation; the death of Patient 1 while awaiting HSCT underscores the severe consequences of delayed diagnosis (Uria-Oficialdegui et al., 2023). Second, given the highly suspected parental gonadal/gonadosomatic mosaicism in this family, particular caution is required when selecting related donors. Even if parental Sanger sequencing of peripheral blood samples is negative, low-level mosaicism associated with shortened telomeres cannot be excluded. Therefore, potential related donors should undergo both *TINF2* variant screening and telomere length assessment (Barade et al., 2022). Finally, because patients with DC exhibit increased sensitivity to alkylating agents and radiation, toxicity-adapted reduced-intensity conditioning (RIC) regimens should be used to reduce transplant-related complications and mortality (Nichele et al., 2023; Pennington et al., 2024). These considerations should be integrated throughout genetic counseling and transplant decision-making for families affected by DC.

Although the *TINF2* p.Thr284Pro (c.850 A>C) variant has been reported, its potential functional impact has not been evaluated *in vitro* (Gupta et al., 2017). In this study, functional assays were performed to explore its potential role. Western blot results showed significantly lower detected TINF2 protein levels in the OE-Thr284Pro group, suggesting that the variant may alter telomere stability through altered interactions with shelterin complex components, although the exact regulatory pathway requires further investigation. CCK-8 assays showed reduced cellular proliferative activity at 48 and 72 h in the mutant group compared with wild-type and OE-CTRL groups, suggestive of impaired cellular proliferation. qPCR showed a reduced relative telomere length signal, consistent with the proband’s findings and with the telomere attrition commonly observed in DC (Khincha et al., 2017). This finding aligns with the known role of *TINF2* as a core shelterin component that stabilizes telomeres through interactions with TRF1, TRF2, and TPP1; mutations disrupt this process (Choo et al., 2022). Moreover, SA-β-Gal staining showed a significantly increased number of blue-stained cells in the mutant group (*P* < 0.001). Senescence-associated β-galactosidase activity and reduced proliferative capacity were observed, consistent with DC pathology (Vittal et al., 2023). *In vivo*, such cellular changes likely affect highly regenerative tissues such as bone marrow, providing insight into the cellular basis of BMF in DC (Choo et al., 2022).

Several limitations should be acknowledged. **First**, functional experiments in this study were performed using HEK293T cells rather than patient-derived hematopoietic stem/progenitor cells, limiting the ability to fully model pathogenic effects within the bone marrow microenvironment. Furthermore, CCK-8 assays only assess cellular proliferation and do not include cell cycle analysis, apoptosis assessment, or telomere dysfunction-induced foci (TIF) measurements. Therefore, the observed proliferation impairment, senescence phenotype, and telomere damage cannot be precisely attributed, and the findings provide only preliminary supportive evidence regarding the deleterious effects of the p.Thr284Pro variant. Second, telomere length assessment relied on TRF and qPCR rather than flow-FISH analysis of leukocyte subsets, which is currently the preferred clinical diagnostic method for telomere biology disorders. This represents a methodological limitation of the present study. Third, Fanconi anemia, an important differential diagnosis for pediatric aplastic anemia, was not formally excluded by chromosome fragility testing (DEB/MMC), representing a limitation in the diagnostic workup. Fourth, the evidence supporting suspected parental gonadal/gonadosomatic mosaicism is indirect and based on the inheritance pattern observed in the family and the absence of the variant in multiple parental tissues. Due to the difficulty of obtaining gamete samples (sperm or oocytes), mosaicism could not be directly confirmed at the gamete level, and the parental origin and exact mosaic fraction of the variant remain undetermined. Fifth, the study included only two affected individuals from a single family, and further validation in additional DC cohorts will be necessary to confirm the generalizability of these findings. Finally, although the mutant construct was associated with lower detected TINF2 protein levels in this overexpression system, and the variant was linked to telomere dysfunction and increased senescence-associated β-galactosidase activity, the downstream molecular mechanisms connecting TINF2 dysfunction to telomere instability remain incompletely understood and warrant further investigation. Moreover, Patient 1, although central to the familial recurrence claim, was less comprehensively characterized than the proband. Telomere length assessment and multi-tissue testing were not performed in Patient 1, limiting the strength of the clinical-genetic comparison between the two affected sisters.

## 5 Conclusion

This study provides the first clinical-genetic observation of the TINF2 c.850 A>C (p.Thr284Pro) variant in two affected sisters with a recurrence pattern compatible with suspected parental gonadal/gonadosomatic mosaicism. Functional analyses provided preliminary supportive evidence that the mutant construct was associated with lower detected TINF2 protein levels in this overexpression system and that this variant may promote telomere dysfunction, increased senescence-associated β-galactosidase activity, and reduced cellular proliferation, the latter potentially reflecting impaired proliferative capacity. Based on the incomplete classical DC phenotype observed in this family, we recommend telomere length assessment and DC-related gene screening in pediatric patients presenting with cytopenia and isolated nail abnormalities, even in the absence of the classic DC diagnostic triad. For suspected telomere biology disorders, age-adjusted telomere length measurement by flow-FISH in leukocyte subsets, combined with germline genetic testing, should be considered the preferred first-line laboratory evaluation. To our knowledge, this is the first report linking this variant to suspected parental gonadal/gonadosomatic mosaicism in a DC family. This conclusion is based on indirect evidence and should therefore be interpreted with appropriate caution.

## Data Availability

The datasets presented in this study can be found in online repositories. The names of the repository/repositories and accession number(s) can be found in the article/Supplementary Material.
