# Supplementary material for: Suspected parental gonadal/gonadosomatic mosaicism for a TINF2 mutation in two sisters with dyskeratosis congenita
Source: Front Genet. 2026 Jul 15;17:1833814. doi: 10.3389/fgene.2026.1833814 (PMC13369613; doi:10.3389/fgene.2026.1833814)
Supplement: Supplementary file 1 [file Table1.docx]

Supplementary Material

**Table 1 ACMG/AMP classification of the TINF2 c.850A>C (p.Thr284Pro) variant.**

| Variant | ACMG Classification | Evidence Codes | Rationale |
| --- | --- | --- | --- |
| *p.Thr284Pro* | Likely Pathogenic (LP) | PM1 + PM2 + PP3 + PP1 + PS3 | **PM1** (moderate, located in the TRF2-binding domain); **PM2** (moderate, absent from gnomAD); **PP3** (supporting, in silico predictions were discordant); **PP1** (supporting, co-segregation in two affected sisters); **PS3** (supporting, functional defects in 293T overexpression, applied at the supporting level due to the non-physiological system). |

**Note**: ACMG classification was performed according to the 2015 ACMG/AMP guidelines (Richards et al., Genetics in Medicine, 2015;17:405–424). Evidence codes: PVS, Very Strong Pathogenic; PS, Strong Pathogenic; PM, Moderate Pathogenic; PP, Supporting Pathogenic; BS, Strong Benign; BP, Supporting Benign. The PS3 evidence for p.Thr284Pro is derived exclusively from the functional experiments conducted in the present study.
