# Supplementary figures and images for: Suspected parental gonadal/gonadosomatic mosaicism for a TINF2 mutation in two sisters with dyskeratosis congenita
Source: Front Genet. 2026 Jul 15;17:1833814. doi: 10.3389/fgene.2026.1833814 (PMC13369613; doi:10.3389/fgene.2026.1833814)

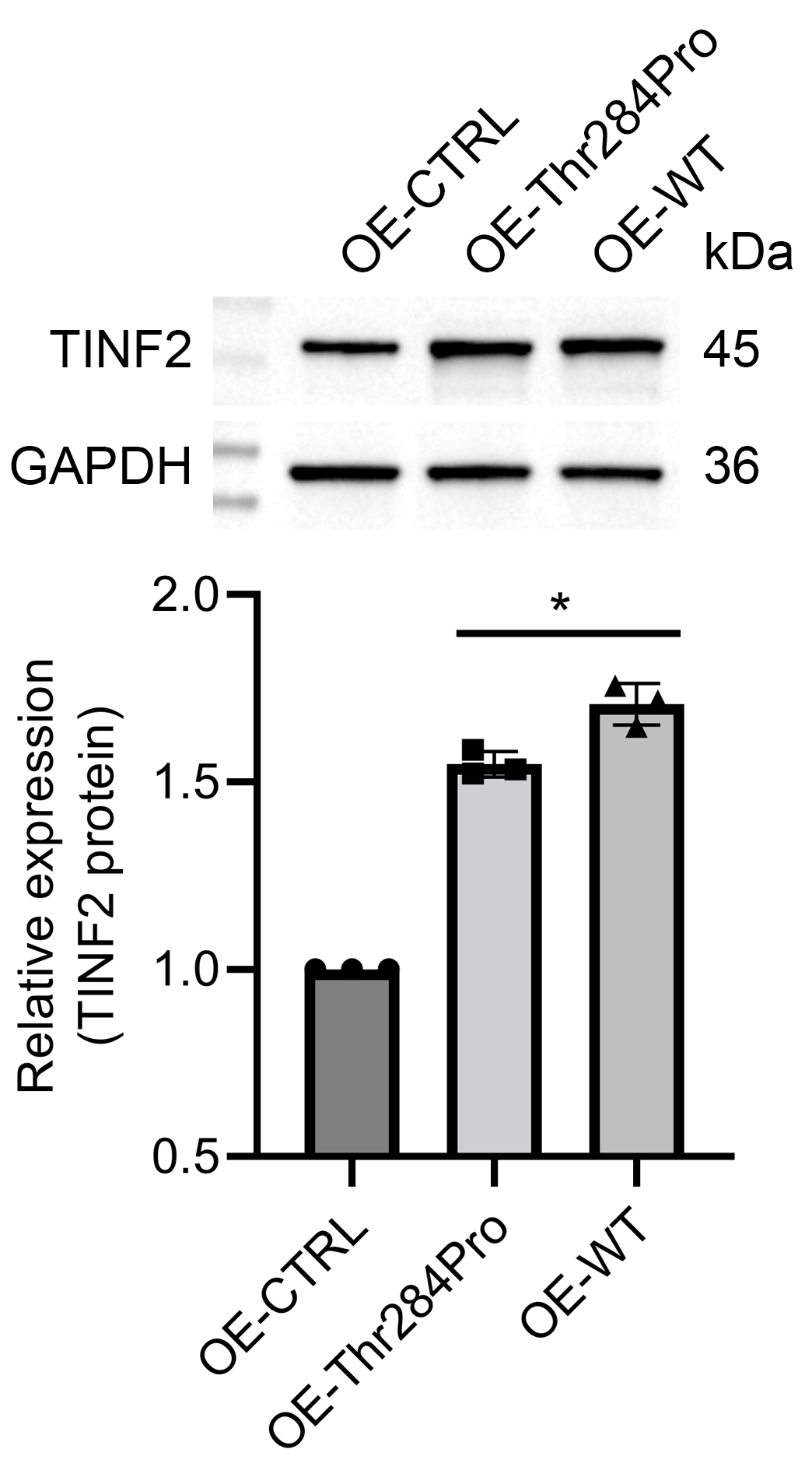

Supplement: Supplementary file 2 [file DataSheet1.ZIP › 整理2.tif]

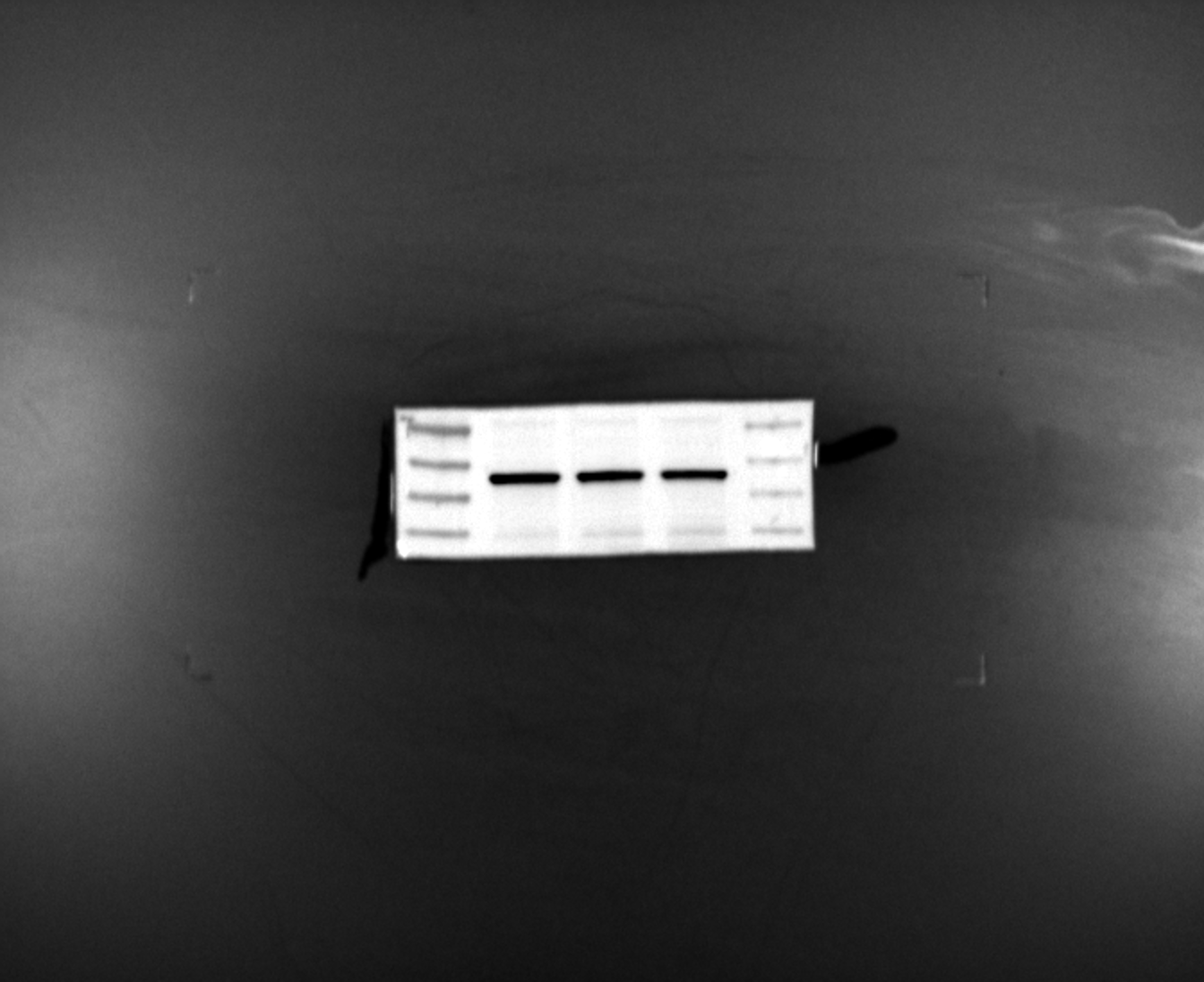

Supplement: Supplementary file 2 [file DataSheet1.ZIP › GAPDH.Tif]

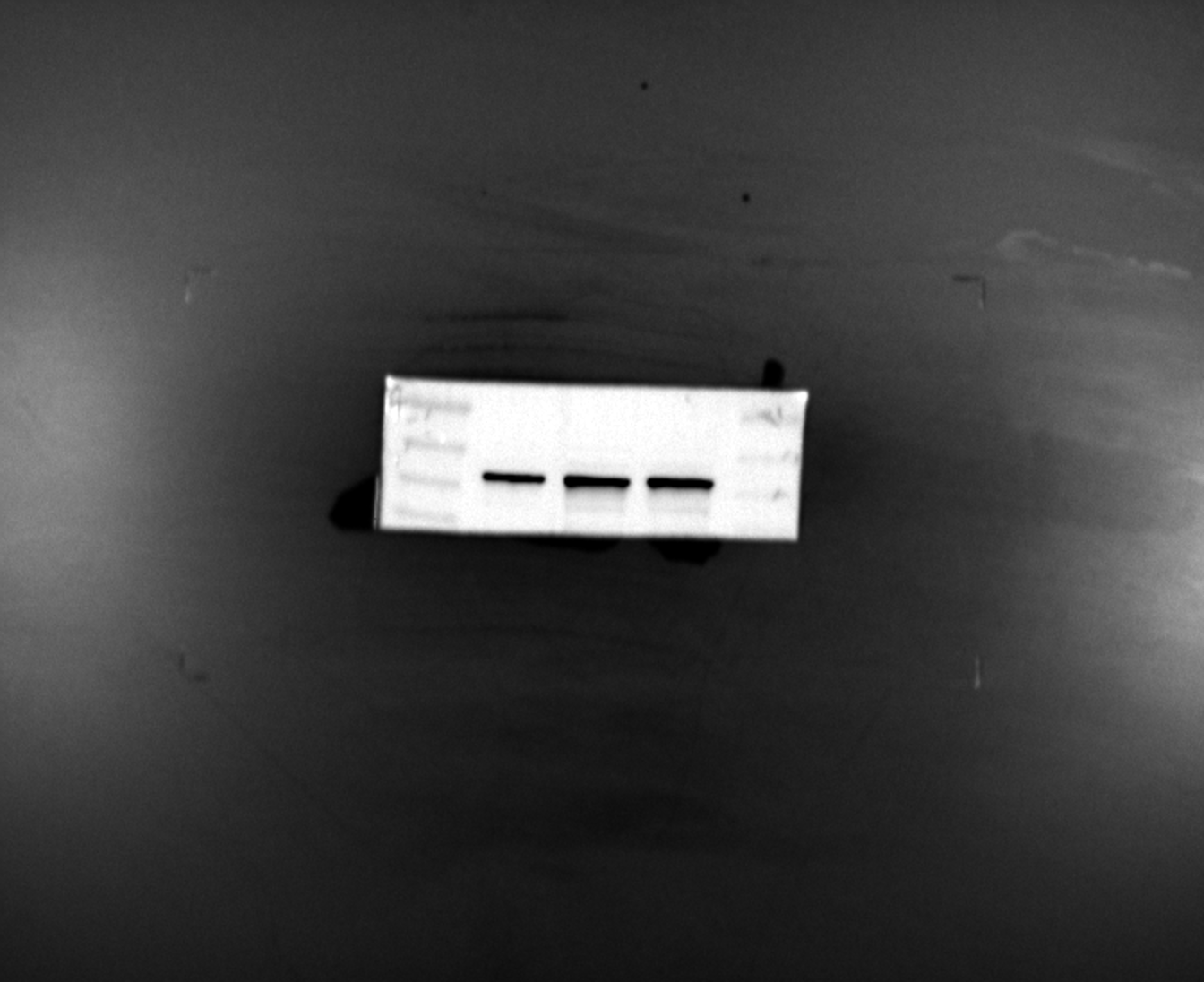

Supplement: Supplementary file 2 [file DataSheet1.ZIP › Tin2.Tif]

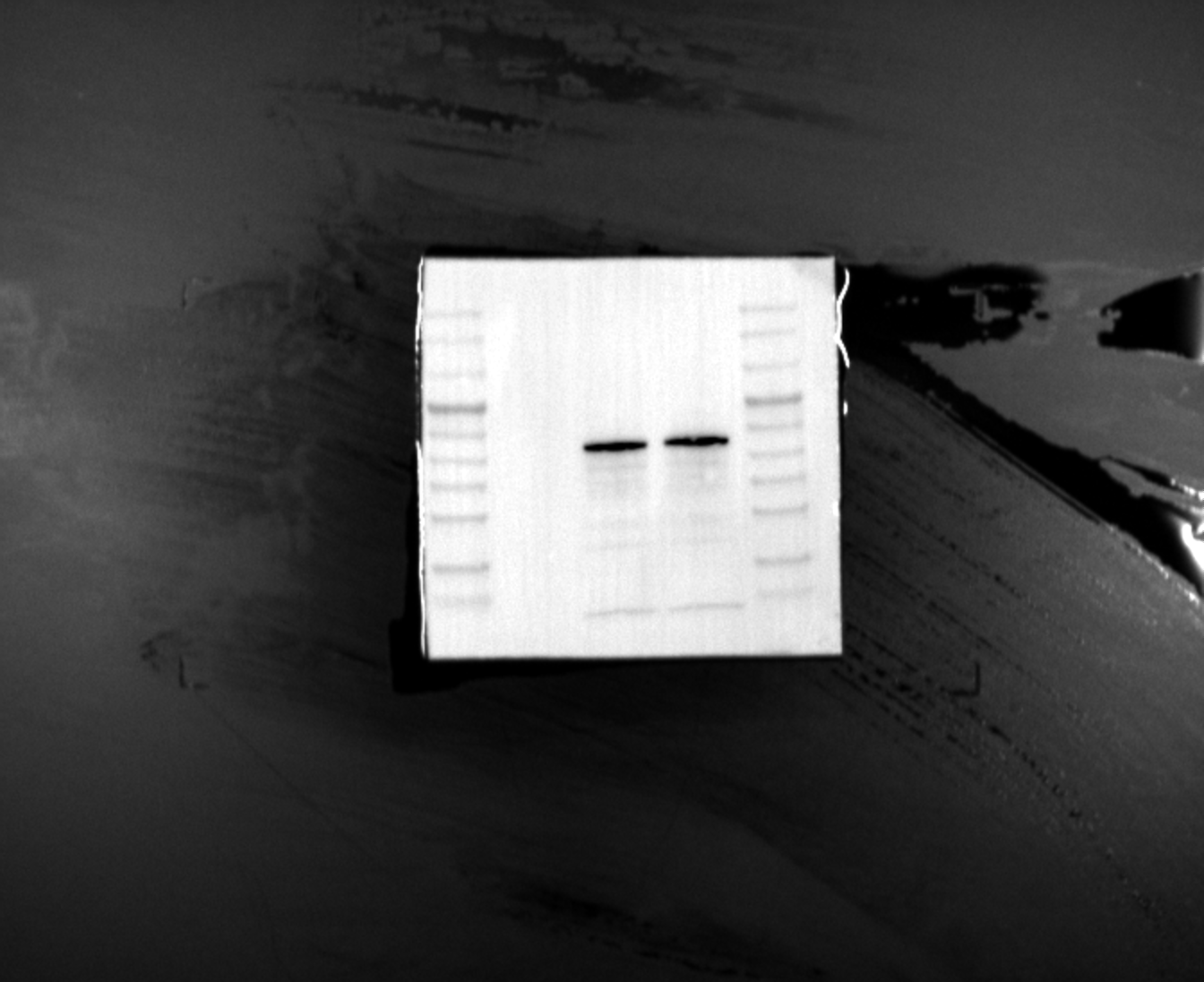

Supplement: Supplementary file 3 [file DataSheet2.ZIP › FLAG.Tif]

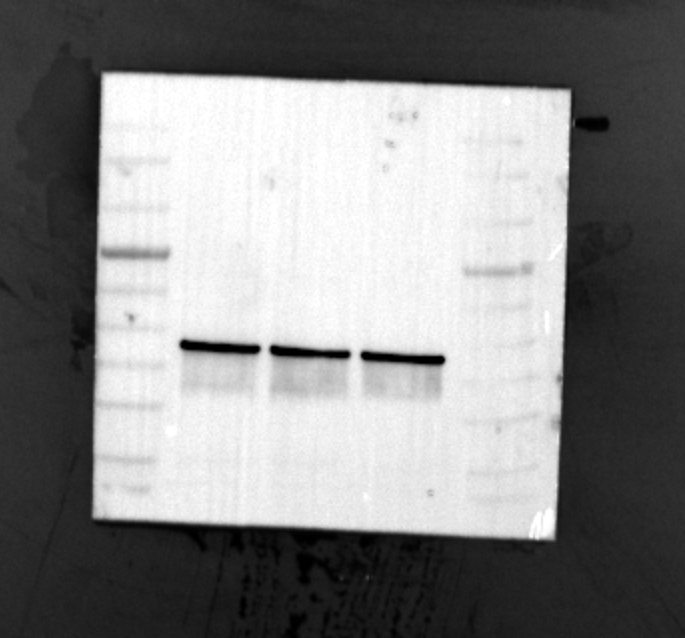

Supplement: Supplementary file 3 [file DataSheet2.ZIP › GAPDH.Tif]
